# Green Synthesis of Silver Nanoparticles Using Natural Extracts with Proven Antioxidant Activity

**DOI:** 10.3390/molecules26164986

**Published:** 2021-08-17

**Authors:** Jolanta Flieger, Wojciech Franus, Rafał Panek, Monika Szymańska-Chargot, Wojciech Flieger, Michał Flieger, Przemysław Kołodziej

**Affiliations:** 1 Department of Analytical Chemistry, Medical University of Lublin, Chodźki 4A, 20-093 Lublin, Poland; 2 Department of Geotechnics, Civil Engineering and Architecture Faculty, Lublin University of Technology, Nadbystrzycka 40, 20-618 Lublin, Poland; w.franus@pollub.pl (W.F.); rapanek@gmail.com (R.P.); 3 Institute of Agrophysics, Polish Academy of Sciences, Doświadczalna 4, 20-290 Lublin, Poland; m.szymanska@ipan.lublin.pl; 4 Chair and Department of Anatomy, Medical University of Lublin, 20-090 Lublin, Poland; wwoj24@gmail.com; 5 Faculty of Medicine, Medical University of Lublin, 20-090 Lublin, Poland; michalflieeeger@gmail.com; 6 Department of Biology and Genetics, Medical University of Lublin, Chodźki 4A, 20-093 Lublin, Poland; przemyslaw.kolodziej@umlub.pl

**Keywords:** green synthesis, silver nanoparticles, antioxidant capacity, reducing power, DPPH scavenging activity, silver nanoparticle antioxidant capacity, CUPRAC, nanoparticle size, zeta potential, SEM, FTIR, UV-Vis

## Abstract

Natural extracts are a rich source of biomolecules that are useful not only as antioxidant drugs or diet supplements but also as complex reagents for the biogenic synthesis of metallic nanoparticles. The natural product components can act as strong reducing and capping substrates guaranteeing the stability of formed NPs. The current work demonstrates the suitability of extracts of *Camellia sinensis*, *Ilex paraguariensis*, *Salvia officinalis*, *Tilia cordata*, *Levisticum officinale*, *Aegopodium podagraria*, *Urtica dioica*, *Capsicum baccatum*, *Viscum album*, and marine algae *Porphyra Yezoensis* for green synthesis of AgNPs. The antioxidant power of methanolic extracts was estimated at the beginning according to their free radical scavenging activity by the DPPH method and reducing power activity by CUPRAC and SNPAC (silver nanoparticle antioxidant capacity) assays. The results obtained by the CUPRAC and SNAPC methods exhibited excellent agreement (R^2^~0.9). The synthesized AgNPs were characterized by UV-vis spectroscopy, Fourier transform infrared spectroscopy (FTIR), scanning electron microscopy (SEM), energy dispersive X-ray analysis (EDX), dynamic light scattering (DLS) particle size, and zeta potential. The UV-vis absorption spectra showed a peak at 423 nm confirming the presence of AgNPs. The shapes of extract-mediated AgNPs were mainly spherical, spheroid, rod-shaped, agglomerated crystalline structures. The NPs exhibited a high negative zeta potential value in the range from −49.8 mV to −56.1 mV, proving the existence of electrostatic stabilization. FTIR measurements indicated peaks corresponding to different functional groups such as carboxylic acids, alcohol, phenol, esters, ethers, aldehydes, alkanes, and proteins, which were involved in the synthesis and stabilization of AgNPs. Among the examined extracts, green tea showed the highest activity in all antioxidant tests and enabled the synthesis of the smallest nanoparticles, namely 62.51, 61.19, and 53.55 nm, depending on storage times of 30 min, 24 h, and 72 h, respectively. In turn, the *Capsicum baccatum* extract was distinguished by the lowest zeta potential, decreasing with storage time from −66.0 up to −88.6 mM.

## 1. Introduction

Nanoparticles (NPs) are of great interest in engineering, chemical, and biomedical sciences. These small particles, due to their high surface-to-volume ratio, have different properties compared to chemical species of the same composition. The differences concern, among other aspects, changes in thermal and electrical conductivity, catalytic activity, optical absorption, melting point, and antibacterial properties. Currently, nanoparticles belong to highly advanced and specialized biomedical products [1]. There are several interesting applications of nanoparticles in medicine and pharmacy: for example, in medical imaging, as nanocomposites, filters, components of drug delivery systems, and formulations for the treatment of cancer [2,3,4,5]. Gold nanoparticles have found applications in analytical procedures for the detection of cancer cells, proteins, and antibodies. Silver nanoparticles are known as antimicrobial agents even against infectious organisms such as *Escherichia coli*, *Bacillus subtilis*, *Vibria cholera*, *Pseudomonas aeruginosa*, *Syphillis typhus*, and *Staphylococcus aureus*. Apart from gold and silver nanoparticles, the literature also describes nanoparticles of zinc oxide (ZnONPs), selenium (SeNPs), copper oxide (CuONPs), copper (CuNPs), nickel oxide (NiONP), and iron (INPs), covering iron oxide (ION) nanoparticles, iron hydroxide (IOOH), and zero-valent iron (ZVI) nanoparticles [6,7,8,9,10,11].

Chemically synthesized nanoparticles often require toxic reducing and stabilizing agents (hydrazine hydrate, sodium borohydride, DMF, and ethylene glycol) [12]. The production of nanomaterials for biomedical applications requires natural methods of synthesis involving microorganisms and biological systems. So-called “green synthesis” or “biogenic synthesis” has been recognized as an ecological approach to the production of a variety of nanomaterials: not only metal/metal oxide nanoparticles but also hybrid materials or so-called bio-inspired materials.

The biosynthesis of nanoparticles can be achieved by reducing metal cations with inactivated tissue, living organisms, or extracts of plants, yeasts, algae, lichens, fungi, and bacteria. The biosynthesized nanoparticles are more stable and less toxic in comparison to those obtained from chemical production. The extract components possessing various bioactive components such as trace metal ions, vitamins, alkaloids, carotenoids, polyphenols, fats, carbohydrates, proteins, biological catalysts (enzymes), etc., play a vital role in nanoparticle formation as strong reducing agents, stabilizing agents, or precursor molecules for NP formation [13,14,15].

The synthesis of nanoparticles takes place in two stages. In the first stage, the metal ions are reduced, and then the agglomeration of colloidal nanoparticles occurs, ultimately forming oligomeric clusters. The main problems encountered in the biogenic synthesis of nanoparticles concern: achieving their appropriate shape, size, and monodispersity in the solution phase. Usually, to control the synthesis process, several factors have been concerned such as pH, temperature, and reaction or incubation time [16,17,18,19].

Various studies have described not only the ability of natural extracts and microorganisms to form AgNPs but also their excellent antioxidant activity, which is higher compared to substrates. It is believed that this activity is due to the preferential sorption of extract components on the surface of nanoparticles [20,21]. Extract-mediated AgNPs have also shown antitumor effects. Asma S. Algebaly et al. [22] have proven the cytotoxicity of AgNPs prepared using *Calligonum comosum* roots and *Azadirachta indica* leaf extracts against LoVo, MDA-MB231, and HepG2 cells. The AgNPs synthesized using *Morinda citrifolia* root extract caused the HeLa cell lines’ total death [23]. In turn, *Alternanthera tenella* [24], *Dendrophthoe falcate*, and *Datura inoxia* [25] extracts appeared to be useful for the preparation of AgNPs inhibiting human breast cancer (MCF-7) cell migration. More recently, the usefulness of *Cannabis sativa* leaf extracts to mediate the green synthesis of AgNPs and their antibacterial activity against several human pathogens (*Escherichia coli*, *Klebsiella pneumoniae*, *Pseudomonas fluorescens*, and *Staphylococcus aureus*) have been proven [26]. An interesting application of plant-mediated AgNPs was demonstrated by Mondéjar-López et al. [27]. The authors obtained AgNPs by the use of *Iris tuberosa* leaf extract. They proved the in vitro antimicrobial properties of AgNPs against typical pathogen contaminants in cosmetics, i.e., *Escherochia coli*, *Pseudomonas aeruginosa*, *Staphylococcus aureus*, *Candida albicans*, and *Aspergillus brasiliensis*. Then, the biogenic-silver nanoparticles were applied as preservative agents in moisturizing cream. Ali et al. [28] also described the anti-candidal activity of AgNPs, biosynthesized using the aqueous leaf extract of *Calotropis gigantean*, which inhibited the growth of *Candida albicans*.

NP preparations owe their therapeutic effects to the synergistic effect of the ingredients as well as a unique bioavailability. Taking into account the variability of NPs obtained by biosynthesis and the richness of natural extracts, there are still opportunities for discovering and developing their new aspects.

The examined natural extracts are well-known sources of specific biomolecules with antioxidant potential [29,30,31,32,33,34,35,36,37,38,39]. All complex endogenous structures contribute to the process of NPs formation. To assess the extract’s ability for nanoparticle synthesis, “Total Antioxidant Capacity” (TAC), which comprises the synergistic and antagonistic effects of components, was measured at the beginning. In the case of multicomponent mixtures, it makes no sense to measure the antioxidant capacity of the individual components, as their isolation and further study are costly and often inefficient. This is particularly important in the case of polyphenols with regard to many unknown representatives of this group. In the case of extracts, the most sensible way to measure their antioxidant capacity is to use a variety of methods that may relate to different mechanisms of antioxidant action [40,41]. The antioxidant evaluation has been conducted using the conventional scavenging free radical method (DPPH), reducing power activity (CUPRAC), and silver nanoparticle-based assay (SNPAC). The results were expressed as equivalents of reference chemicals such as chlorogenic acid, ascorbic acid, and Trolox. Then, the green synthesis of AgNPs was performed. Obtained NPs have been characterized by SEM, FTIR, and UV-vis spectroscopy. The comparison of the antioxidant properties of extracts with the features of extract-mediated AgNPs allowed for the assessment of their suitability for the green synthesis of NPs with defined parameters.

## 2. Results

### 2.1. Determination of Antioxidant Potential of Examined Extracts

Many in vitro tests are used to measure antioxidant activity that are based on the mechanism of hydrogen transfer (HAT) or electron transfer (ET) from the antioxidant to free radicals. Recently published reviews detail the usefulness and principles of these procedures [42,43]. Most authors agree that the antioxidant activity of natural samples should be tested with several methods adapted to the purpose of the research. In the case of our research, the methodologies used to assess the antioxidant capacity include an electron transfer mechanism, as the formation of nanoparticles depends primarily on the reducing capacity of the extracts.

The electron transfer (ET) assays include the Folina–Ciocalteu (FC) test, phosphomolybdenum assay, ferrous oxidation-xylenol orange assay (FOX), iron antioxidant power reduction (FRAP), chromium reducing antioxidant capacity (CHROMAC), copper antioxidant capacity reduction test (CUPRAC), or Ce (IV)-based reducing capacity (CERAC). These are color tests; therefore, spectroscopic techniques can be used for measurement. The change in absorbance level is linearly correlated with the total antioxidant capacity of the sample. From this group of tests, the CUPRAC test, which is commercially available in a microplate version, was selected for testing.

Nanotechnology tests are also classified as electron transfer tests [44]. In our study, the silver nanoparticle antioxidant capacity (SNPAC) test developed by Özyürek et al. [45] was used.

There are also tests that operate in mixed mode (HAT/SET) such as DPPH (2,2-diphenyl-1-picrylhydrazil, ABTS (2.20-azinobis-3-ethylbenzthiazolin-6-sulfonic acid), or DMPD (N, N-dimethyl-p-phenylenediamine dihydrochloride). In these tests, a stable free radical such as DPPH^●^ or cationic radicals such as DMPD^+●^ and ABTS^+●^ are scavenged with an antioxidant. Despite the fact that the DPPH assay is usually classified as a mixed-mode (ET/HAT) assay, there is some evidence that the ET mechanism dominates in non-aqueous solutions due to the ability of organic solvents to form strong hydrogen bonds with antioxidants [46,47]. Prepared methanolic extracts, rich in molecules with various antioxidant properties, have the ability to change the oxidation level of metal ions by supplying electrons. Therefore, methods based on the electron transfer mechanism were chosen to determine the total antioxidant capacity (TAC). DPPH, CUPRAC, and SNPAC methods allow for initially estimating the suitability of the prepared extracts for electron delivery and the rate of AgNP formation.

#### 2.1.1. Reducing Power Activity by CUPRAC Assay

The CUPRAC Antioxidant Assay Kit was used to determine the total antioxidant capacity (TAC) of examined extracts. In the first step, calibration curves were constructed for the reference substances such as chlorogenic acid, ascorbic acid, and Trolox. The linear regression analysis parameters evaluated for the standard curves are summarized in Table 1. An excellent linearity in the range from 300 to 1000 μm was achieved. The determination coefficient exceeded 0.99. Based on the regression equations and measurements made for the tested extracts, the equivalent values corresponding to each standard antioxidant were calculated (Table 2).

The reduction of metal ions, e.g., Cu (II), is commonly used as a test to assess the antioxidant activity of natural extracts. The reduction ability is a measure of their electron donating properties. The obtained results were expressed as equivalents of reference substances. It is known that the higher the equivalents, the higher the reducing activity of the test sample. Generally, the highest activity in the CUPRAC test was obtained for *Camellia sinensis* and *Ilex paraguariensis* extracts, and the lowest for *Porphyra Yezoensis*, a representative of marine algae. It has been shown many times that the high reduction potential of copper is associated with the high level of phenolic compounds in the extracts, which justifies the above result. Such a relationship was confirmed in the studies conducted by GonÇalves et al. [48] and Srinivasan et al. [49]. Moreover, Zengin et al. [50], on the example of *Asphodeline anatolica* extracts, showed that the reduction ability is higher in methanol than in aqueous extracts.

Thus, the preparation of methanol extracts in order to enhance the reduction potential seems to be a reasonable choice. This can also inspire further research to expand the range of extraction solvents.

#### 2.1.2. DPPH Free Radical Scavenging Activity

The DPPH owing to a stable nitrogen radical was very early adapted to study the free-radical quenching abilities of different antioxidants [51]. Despite its simplicity, the DPPH assay possesses some problematic issues such as varied reaction kinetics with different antioxidants or the reversibility of the free radical scavenging reaction. Despite these disadvantages, the test is still commonly applied for examining the antioxidant activity of natural extracts [43].

In the first step, calibration curves were performed for solutions of reference substances: ascorbic acid, chlorogenic acid, and Trolox. Measurements were performed for six different concentrations of standard solutions containing DPPH at a constant concentration of 0.1 mM at 517 nm after 15 min storage in a dark place, considering the previously confirmed fast reaction kinetics between DPPH and the chosen antioxidant standards [52]. The calibration plots were subjected to simple linear regression analysis. The parameters of the generated equations are listed in Table 3.

The antioxidant activity of methanolic extracts was tested under uniform conditions, taking into account the optimized DPPH concentration of 0.1 mM at 517 nM. The reaction time was set at 60 min assuming the differentiated extracts’ components with possible slow kinetics. The addition of an increasing volume of the extract caused a clear change of the color of DPPH from purple to yellow. In order to compare the antioxidant properties of the tested samples, the obtained % I values were converted into equivalents of reference substances, using the appropriate calibration equation. The results are collected in Table 4.

The DPPH scavenging activity of the examined extracts varied from poor (algae) to very good scavengers (green tea). Generally, the order of extract activities was similar to that established by the CUPRAC assay. The free radical scavenging activity of extracts can be arranged in the following order: *Camellia sinensis* > *Tilia cordata* > *Ilex paraguariensis* > *Salvia officinalis* > *Aegopodium podagraria* > *Levisticum officinale* > *Urtica dioica* > *Viscum album* > *Capsicum baccatum* > *Porphyra Yezoensis*. The antioxidant capacity of extracts is mainly attributed to the redox potential of phenolic compounds, acting as reducing agents. As it was mentioned before, DPPH can accept an electron as well as a hydrogen radical, becoming a stable diamagnetic molecule.

#### 2.1.3. Silver NanoParticle Antioxidant Capacity (SNPAC) Measurements

In 2006, Scampicchio et al. [44] described a method based on the catalytic growth of gold (Au) nanoparticles. Several years later, Özyürek et al. [45] developed a sensitive colorimetric method consisting of the reduction of Ag ions to silver nanoparticles (SNP) for the detection of polyphenols. The increase in SNP on monodisperse seed particles prepared by reducing Ag ions by the use of trisodium citrate was due to the addition of antioxidants as secondary reducing agents. The stable suspension containing spherical silver nanoparticles (SNPs) exhibited a very intense surface plasmon resonance (SPR) absorption band at 423 nm. The method was named by the research group “silver nanoparticle antioxidant capacity” (SNPAC). This sensitive colorimetric method is recommended for measuring the total antioxidant capacity (TAC) by its precursors for the investigation of complex samples [45] of plant samples containing polyphenols (i.e., flavonoids, simple phenolic, or hydroxycinnamic acids). Despite the proven capability of natural samples to form AgNPs as sensitive antioxidant probes, the method has only been used in a few studies [53]. In the recorded spectrum, one can see an increase in absorbance at 423 nm with increasing antioxidant concentration. The creation of AgNPs was visible as a yellowish-brown color in an aqueous solution due to the excitation of surface plasmon vibrations in silver nanoparticles [52,54].

**Figure 1:**
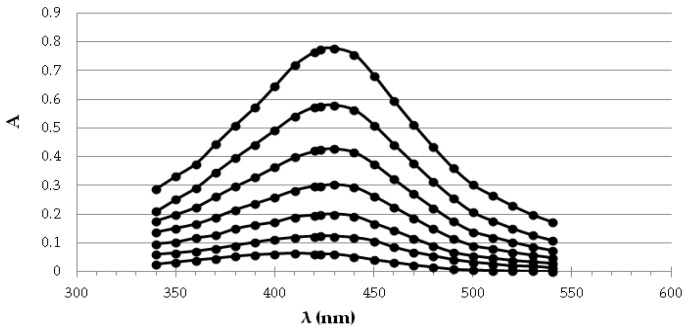
AgNPs absorption spectra measured spectrophotometrically in the range of 340–540 nm, obtained with increasing concentration of chlorogenic acid from the bottom to the top: 0; 3.5; 7.14; 10.71; 14.25; 17.85; 25 µM).

The exemplary spectra recorded for different dilutions of the chlorogenic acid standard are presented in Figure 1. Under the influence of the increasing concentration of the reference antioxidant, the enlargement of the embryos takes place, which is reflected in the systematic increase in absorbance. The calibration curves were made for each standard by measuring the absorbance of the dilution series at 423 nm. The obtained curves were subjected to regression analysis (Table 5). The method gave an excellent linear response over a wide concentration range of standards (R^2^ > 0.9). The limits of detection (LODs) for standards in the SNPAC assay were from 1.51 to 5.85 µM.

**Figure 2:**
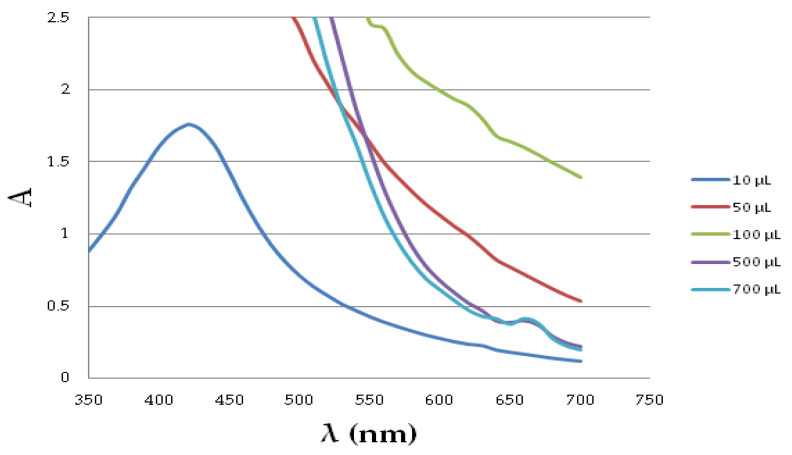
Spectra measured after 30 min in dark place of SNP solution mixed with different volumes of *Camellia sinensis* extract (10, 50, 100, 500, 700 µL).

To estimate the antioxidant activity of examined extracts, different volumes of extracts were added to the SNPs solution. Figure 2 presents the influence of the extract volume on the shape of the spectra in the range of 350 nm–700 nm on an example of *Camellia sinensis* extract. As it can be seen, the increase in the volume of the extract in the test sample resulted in an increase in absorbance even beyond the measurable range. The visible wide absorption band in the UV-vis spectrum in the range of ~400–450 nm is characteristic of silver nanoparticles. One of the observed effects of the plasmon resonance of the formed nanostructures was an intense color that changes from straw yellow to dark brown. As the volume of the extract increases from 100 to 700 µL, an additional slight absorption band in the red light range appeared between 650 and 700 nm. Thus, in the case of larger volumes of the extract, i.e., 500 and 700 µL, the possibility of the agglomeration of nanoparticles should be considered. Such an effect can also be related to the possible formation of rod-shaped nanoparticles. In this case, two absorption bands appear on the spectrum: one caused by electron oscillations along the structure, and the other related to vibrations at its ends [55]. If the nanoparticle has a large length/width ratio, it is possible to shift the plasmon resonance into the infrared spectral range.

**Figure 3:**
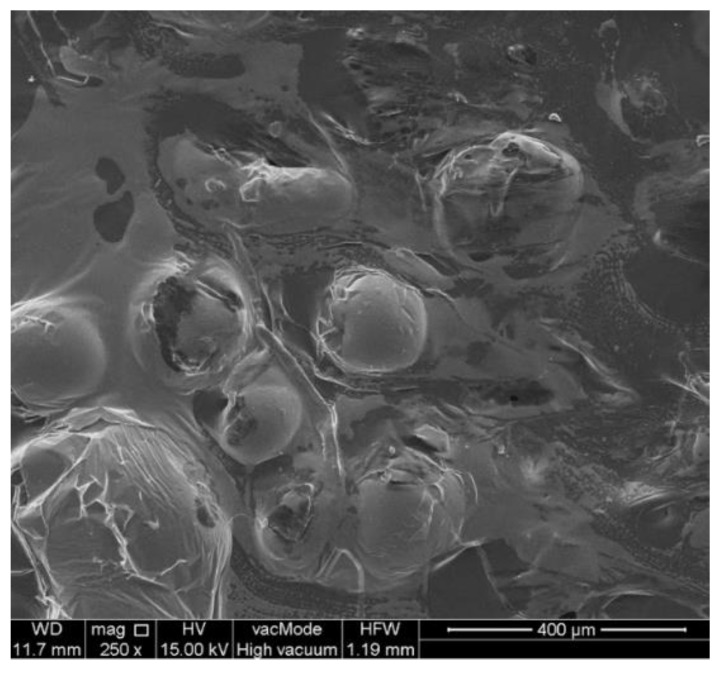
SEM image of silver nanoparticles produced in SNPAC method by 100 µL of *Tilia cordata* extract.

The increasing absorbance value confirmed the ongoing intensive bioreduction process along with the increasing concentration of the extract. Photographs of the obtained spherical nanoparticles made by SEM microscopy show that the nanoparticles are “suspended” in a gelatinous organic substance (Figure 3) acting as a stabilizer of the resulting nanoparticles.

The antioxidant activity of the remaining extracts was measured utilizing appropriate volumes (10, 50 µL) to allow spectrophotometric reading. The measurement results were used to determine the equivalents of the reference substances on the basis of the calibration equations (Table 6). Comparing the results, the trend of the total antioxidant power reflecting the cumulative action of all sample constituents can be arranged as follows: *Camellia sinensis*, *Ilex paraguariensis*, *Salvia officinalis*, *Tilia cordata*, *Levisticum officinale*, *Aegopodium podagraria*, *Urtica dioica*, *Capsicum baccatum*, *Viscum album*, and *marine algae*.

### 2.2. Comparison of Methods for Antioxidant Potential Evaluation

The results of the reference substance equivalents obtained for the individual extracts differ significantly from one another. Nevertheless, the TAC equivalents found by the microdilution CUPRAC method in 96-well plates correlate well with those of the SNAPC. The correlations obtained are described by the following equations generated separately for the individual standards of chlorogenic acid (CAeq.), ascorbic acid (AAeq), and Trolox (Txeq):

Tx_eq_ (CUPRAC) = 1.3702 Tx_eq_(SNAPC) − 6.5971, R^2^ = 0.9041 (1)

AA_eq_(CUPRAC) = 3.3277 AA_eq_ (SNAPC) − 5.8117, R^2^ = 0.9047 (2)

CA_eq_(CUPRAC) = 2.8859 CA_eq_ (SNAPC) − 3.2232, R^2^ = 0.8963 (3)

The highest equivalent capacities in the SNPAC method were observed for green tea and Yerba mate. The huge antioxidant potency of these extracts is caused by the catechin flavonoids, known as “tea antioxidants”, owing to the presence of multiple OH functional groups in their structures. The detection limits (LOD) for the reference substances (ascorbic acid, chlorogenic acid, and Trolox), determined by the above methods, differed significantly from each other. In general, the most favorable values, and thus the smallest ones, were found for all reference substances in the DPPH free radical scavenging method (0.8498–1.8644 µM). An order or even two orders of magnitude of higher values were obtained for the CUPRAC method (65.48–115.23 µM). In turn, the LOD values obtained by the SNPAC method range from 1.5141 µM to 5.8548 µM.

### 2.3. Characteristics of AgNPs Obtained by Biogenic Synthesis with Examined Natural Extracts

#### 2.3.1. UV-Vis Spectroscopy

Spectrophotometric spectra of the synthesized nanoparticles were measured after 30 min, 24 h, 72 h, and 96 h of their storage in a dark place. The absorbance at 423 nm, which is characteristic of silver nanoparticles, increases with the storage time. The increase in absorbance was also visible to the naked eye as a darkening of the solution. The greatest increase in absorbance occurs after 96 h. The observed increase (∆Abs = Abs_96h_ − Abs_30min_) can be arranged in the following order: *Camellia sinensis* (3.36), *Salvia officinalis* (1.75)*, Tilia cordata* (0.95), *Aegopodium podagraria* (0.92), *Urtica dioica* (0.62), *Capsicum baccatum* (0.58), *Levisticum officinale* (0.50), *Viscum album* (0.03), *Ilex paraguariensis* (0.01)*,* and *Porphyra Yezoensis* (0.0). The current results are in agreement with the literature [56,57,58]. Considering other reports on the synthesis of NPs, Mock et al. [55] reported the absorbance peak at 420, 430, and 435 nm for AgNPs synthesized using *Boswellia ovaliofoliolata*, *Shorea tumbuggaia*, and *Svensonia hyderobodensia*. Bala et al. [58] confirmed AgNPs synthesized by *Aspergillus fumigates* by surface plasmon resonance at 450 nm. Sriramulu [56] detected absorbance peaks at 434 and 422 nm for AgNPs synthesized by using forest and edible mushrooms. In another study, silver nanoparticles formed in different plant leaf extracts possessing round shapes exhibited surface plasmon resonance peak wavelengths between 422 nm and 451 nm [59].

#### 2.3.2. FTIR Spectroscopy

**Figure 4:**
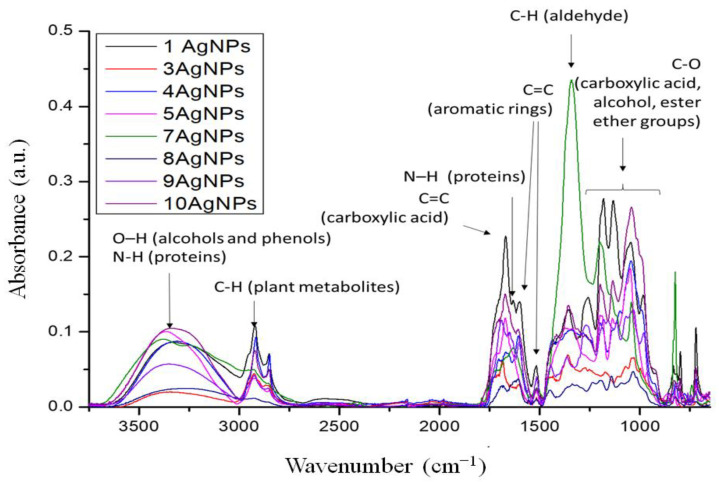
FTIR spectra in the range 4000–650 cm^−1^ of AgNPs synthesized in presence of extracts from: 1-*Aegopodium podagraria*, 3-Salvia officinalis, 4-*Tilia cordata*, 5-*Viscum album*, 7-*Porphyra Yezoensis*, 8-*Camellia sinensis,* 9-*Ilex paraguariensis*, and 10-*Levisticum officinale*.

The FTIR spectra of the AgNPs were recorded to identify the functional groups involved in the synthesis of AgNPs (Figure 4). The spectra, apart from the synthesized AgNPs, also contained the plant extract components. Unfortunately, two samples based on extracts from 2-*Urtica Dioica* and 6-*Capsicum baccatum* were not measured due to the impossibility of freeze-drying of these samples. The overall observation from the FTIR bands (Figure 3) confirmed that the bioactive component is in charge of the reduction of silver ions to AgNPs. The band at 3304–3340 cm^−1^ is assigned to the stretching vibration of the OH group, while the band at 2923 cm^−1^ indicates C-H stretching. The most promising bands that can be responsible for the reduction and stabilization of formed AgNPs are the bands connected with the vibration of functional groups, such as alcohols, phenols, carboxylic acids, etc. The band around 1620 cm^−1^ is due to the amide C=O stretch. The band with a maximum between 1350 and 1400 cm^−1^ can be assigned for the alkane -C-H- bond. The band at 1000–1050 cm^−1^ represents the C-O stretching of carboxylic acids, alcohols, esters, and ether groups [60].

**Figure 5:**
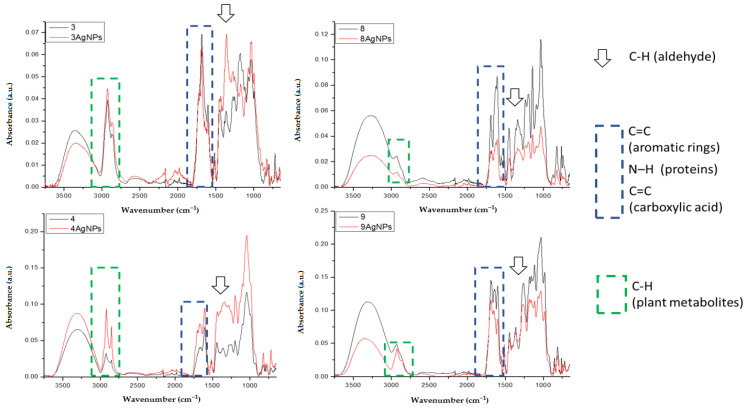
FTIR spectra in the range 4000–650 cm^−1^ of freeze-dried extracts and AgNPs synthesized in presence of extracts from: 3-*Salvia officinalis*, 4-*Tilia cordata,* 8-*Camellia sinensis,* and 9-*Ilex paraguariensis*.

Additionally, the comparative spectra of pure extracts and extracts with synthesized AgNPs were obtained for chosen samples: 3-*Salvia officinalis*, 4-*Tilia cordata,* 8-*Camellia sinensis,* and 9-*Ilex paraguariensis*. The bands that are most significant/intense for taking part in the synthesis and stabilization of AgNPs are presented in Figure 5. These bands are connected with C-H from aldehyde (1338 cm^−1^), bands C=C from aromatic rings and/or carboxylic acids, N-H from proteins (1730–1560 cm^−1^), and C-H from plant metabolites (2980–2770 cm^−1^). These results are in agreement with the investigation of Balaji Venkatesan et al. [61] who reduced silver ions in the presence of extracts from *Rosa damascene*. Manikandan et al. [62], who reduced Ag ions by *R. indica* extract, indicated that the band at 1624 cm^−1^ shifted to a higher wavelength, confirming the formation of AgNPs.

#### 2.3.3. Scanning Electron Microscopy

**Figure 6:**
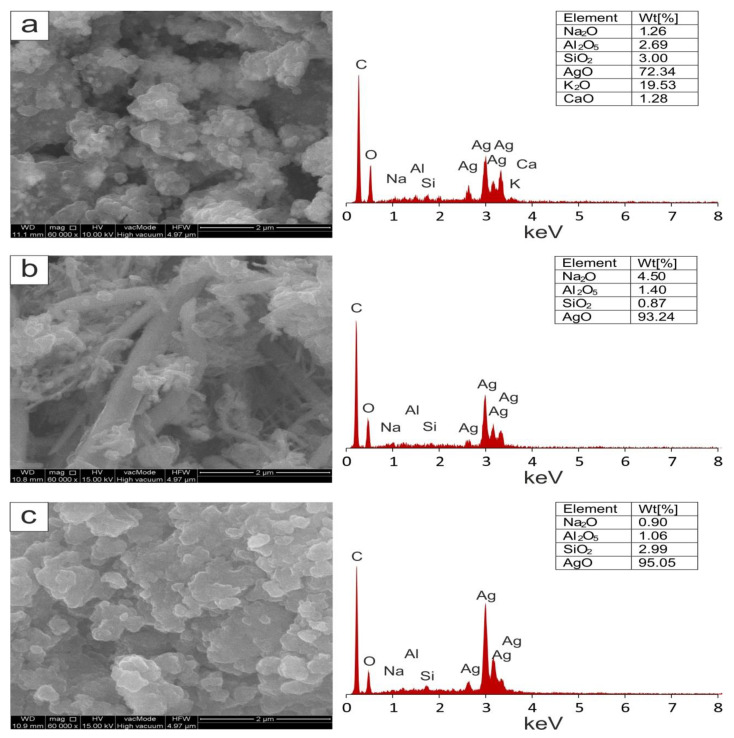
SEM-EDX spectra recorded from NPs synthesized by *Aegopodium podagraria* (**a**), *Salvia officinalis* (**b**), and *Viscum album* (**c**) extracts.

The scanning electron microscopy (SEM) technique was applied with the aim to visualize the shape of silver nanoparticles. The representative SEM images including various forms of extract-mediated silver nanoparticles are presented in Figure 6. The shapes of extract-mediated AgNPs were mainly: (i) spherical, which have a diameter of about 100–200 nm, and their aggregates reach a size of up to 2 µM which was observed for *Aegopodium podagraria* (Figure 6a); (ii) rod-shaped of varying sizes—the larger ones reach lengths up to 5 µM and diameters of 0.4 µM, distributed irregularly, whereas smaller ones often form grid-like aggregates, as was observed for *Salvia officinalis* (Figure 6b); and (iii) irregularly shaped plates with slightly rounded edges that are up to 2 µM for *Viscum album* (Figure 6c). Considering the same synthesis conditions, it can be concluded that morphological features of AgNPs are affected mostly by the kind of extract used. The applied sample preparation methodology (freeze-drying) can also influence the visible aggregation of nanoparticles. The presence of silver in samples was confirmed by the observation in EDS spectra in which there are characteristic signals at 2.62, 2.98, 3.14, 3.34, and 3.50 keV. The presence of other elements (Na, K, Ca, Al, Si) on the EDS spectra is related to the chemical composition of the plant matrix.

#### 2.3.4. Zeta Potential and Size of AgNPs

**Figure 7:**
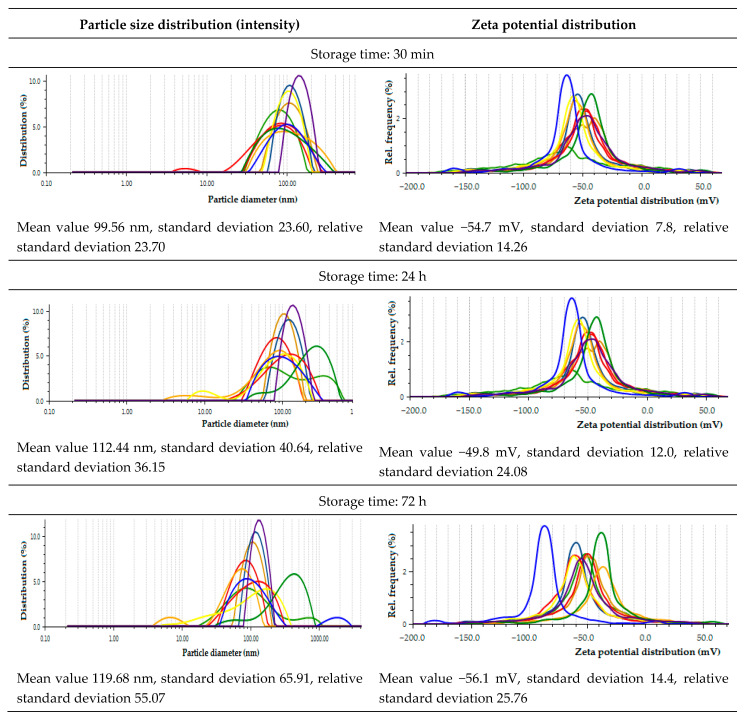
Zeta potential and hydrodynamic particle diameter of biosynthesized AgNPs depending on the time of incubation (30 min, 24 h, 72 h). Conditions: the ratio of 1 mM AgNO_3_ to extract was 30:2 (*v*/*v*). Legend: *Camellia sinensis* (light red), *Ilex paraguariensis* (dark orange)*, Salvia officinalis* (yellow)*, Tilia cordata* (dark green)*, Levisticum officinale* (dark blue)*, Aegopodium podagraria* (light green)*, Urtica dioica* dark red)*, Capsicum baccatum* (light blue)*, Viscum album* (light orange), and *Porphyra Yezoensis* (violet).

Analyzing the effect of the storage time on the size and zeta potential of extract-mediated nanoparticles, the following trends could be observed (Figure 7): as time passes from 30 min to 72 h, the mean value of the hydrodynamic diameter underwent elevation from 99.56 to 119.68 nm. The coefficient of variance of the AgNPs, being the ratio of SD (standard deviation) to average hydrodynamic diameter, was in the range of 0.23–0.55. The largest nanoparticles were further enlarged; for example, *Tilia cordata* extract-mediated NPs increased their size from 88.41 to 2946 nm. In turn, the smallest ones reduced their size with time, such as *Camellia sinensis* extract-mediated NPs for which the beginning size of 62.51 nm depressed to 53.55 nm at 72 h after synthesis. This process can be compared to the maturation of crystalline sediments when their size is normalized as a result of dissolving small and growing large forms. It is significant that the smallest nanoparticles were obtained for the green tea extract, for which the measured antioxidant potential was one of the highest. Moreover, it can be seen that at the beginning, the population of nanoparticles is more homogeneous, while after 72 h, it diversifies. The zeta potential oscillated around −53.5 mV (−54.7; −49.8; −56.1 mM), indicating the sorption of the charged extract components, which stabilize the NPs and counteract aggregation. It should be emphasized that the NPs that were synthesized with *Capsicum baccatum* extract showed the most negative zeta potential. In this case, the zeta potential becomes increasingly negative over time, decreasing from −66.0 mV to −88.6 mM. Recent studies concerning the biosynthesis of AgNPs with *Capsicum* extract indicated that the proteins which have amine groups are responsible for the reduction during the formation of silver nanoparticles. It was proven that the secondary structure of the proteins is able to change after reaction with silver ions [54,63].

## 3. Discussion

The biogenic synthesis of metallic nanoparticles with the use of extracts of natural plants, bacteria, fungi, lichens, etc., is widely described in the literature [64]. The synthesis of metallic NPs using plant extracts is gaining more and more supporters due to the fact that it provides environmentally friendly conditions. It turns out that not only does it not generate toxic waste, but it also protects against unfavorable contamination of the final product, which enables its use in therapy as an antibacterial substance, carrier of medicinal substances, or contrasting reagent in medical diagnostics (MRI). The still-growing popularity of this method is determined not only by ecological but also economic considerations due to the easy and almost cost-free availability of raw materials, for which, e.g., agro-industrial waste materials are often used [65,66]. Initially, biogenic syntheses of nanoparticles was carried out with the use of selected model compounds, mainly phenolic acids, due to their wide occurrence and high reducing power [67]. Extracts prepared from natural raw materials, however, have a complex composition, which is the source of many phytochemicals, acting not only as reducing agents but also as stabilizing factors that ensure the appropriate monodispersity of nanoparticles. All antioxidants and complex endogenous structures present in the sample take part in it; therefore, the process of generating NPs by green synthesis is not selective. There are many reports in the literature on the suitability of individual raw materials for the green synthesis of nanoparticles [43]. However, it is difficult to compare them with each other, mainly due to the different conditions of extraction or synthesis. Apart from a few exceptions [68], there is still a lack of studies that make it possible to compare different raw materials under uniform experimental conditions. Therefore, the present study was undertaken to compare the possibilities of ten different extracts, prepared under the same conditions from readily available natural raw materials, rich in, among other materials, phenolic compounds, for the production of AgNPs.

In order to assess the potential of the extracts to reduce silver ions, their antioxidant activity was determined using three independent methods (DPPH, CUPRAC, SNPAC), where electron transfer is the dominant mechanism and key for green synthesis. The results of the antioxidant activity were presented in the form of the equivalents of the reference substances of ascorbic acid, chlorogenic acid, and Trolox. The methods’ sensitivity can be arranged according to LOD values, estimated by the 3 σ procedure, in the following order: DPPH (0.8498–1.8644 µM) > SNAPC (1.5141–5.8548 µM) > CUPRAC (65.48–115.23 µM). A very good correlation was confirmed between the results estimated by the CUPRAC and SNPAC methods (R^2^~0.9). A similar correlation between the AgNP-based method and the cupric ion-reducing applied for the evaluation of the total antioxidant activity of polyphenols was reported previously by Özyürek et al. [45]. The authors declared the determination coefficient of the above relationship on the level of R^2^ = 0.8761. Azat Akbal et al. [59] also observed a strong relation of the AgNP formation rate and CUPRAC antioxidant capacity of *Punica granatum*, *Cydonia oblonga*, *Castanea sativa*, *Ficus carica*, *Juglans cinerea*, *Morus nigra*, and *Morus alba* aqueous leaf extracts.

The obtained results showed that methanol extracts with a confirmed, differentiated antioxidant potential ensured the efficient synthesis of AgNPs. The mean size of the obtained nanoparticles ranged from 99.56 to 119.68 nm, changing with time from 30 min to 72 h. Green tea extract deserves special attention, as it showed the highest antioxidant activity in all tests and enabled the synthesis of the smallest nanoparticles, namely 62.51, 61.19, and 53.55 nm, depending on storage time. This observation confirms earlier findings that the size of the AgNPs formed may vary depending on the type of reducing agents and stabilizers of the reducing reaction [69]. In general, the greater the reduction potential, the faster the reaction rate and the production of smaller nanoparticles. The latest review of Teixeira et al. [70] also highlighted the extraordinary effectiveness of *Camellia* species as natural antioxidants. It should be emphasized that the impressive antioxidant potency of *Camellia* extract is caused by the polyphenolic compounds, mainly (–)-epigallocatechin-3-gallate, (–)-epigallocatechin, (–)-epicatechin-3-gallate, and (–)-epicatechin, owing to the presence of multiple OH functional groups in their structures. As the research conducted so far shows, it is the hydroxyl groups, especially those located in the ortho and para positions, that are involved in the synthesis of metal nanoparticles [71,72]. Litvinenko and Ingold proved that the 7-OH group in flavonoids also plays a very important role as a site of ionization and electron transfer according to the SPLET mechanism [73,74].

The influence of elevated temperature on the kinetics of nanoparticle formation has already been investigated. It is known that at a higher temperature, even exceeding 100 °C, nucleation usually dominates over the growth of nanoparticles, which results in a reduction of their size [75,76]. In our study, we showed that both the size and the value of the surface charge of nanoparticles change with storage time. In our study, *Tilia cordata* extract-mediated NPs increased by almost 2.5 times in size within 3 days after synthesis, whereas the smallest NPs obtained with the *Camellia sinensis* extract decreased by about 15%. The process is similar to the maturation of crystalline sediments when their size is normalized, as small forms disappear and larger ones expand. A similar effect was observed by Dwivedi et al. [77] who performed AgNP synthesis using white quinoa leaf extract. Based on the intensity of the UV-vis spectra, they observed the progress of the reaction over time, up to 2 hours after synthesis. The zeta potentials in our study were smaller than −35 mV, indicating the existence of an electrostatic stabilization of nanoparticles. However, we cannot exclude the existence of steric or electro-steric stabilization due to the absorption of biomolecules on the NPs’ surface enhancing during the prolonged storage time. 

In turn, the *Capsicum baccatum* extract was distinguished by the lowest zeta potential. It is known that paprika extract is a source of phenolic compounds such as quercetin, capsaicinoids (mainly capsaicin), and polyphenolic compounds, including flavonoids (e.g., luteolin-7-glucoside), carotenoids (capsanthin, β-carotene), and vitamin E, and C [78,79], which are responsible for antioxidant activity. All of these compounds take part in the bioreduction process. However, in stabilization, which plays an essential role in ensuring the dispersion of the suspension, in addition to compounds containing carboxyl groups, proteins and carbohydrates, which are the dominant components, are mainly involved. FTIR studies confirmed the participation of carboxylic acids, alcohol, phenol, esters, ethers, aldehydes, alkanes, and proteins in the reduction and stabilization of the AgNP suspension.

The increase in the volume of the extract caused an increase in the intensity of the SPR band, but in such conditions, an additional band appeared shifted towards longer wavelengths. The reasons for this may be different, but they are usually not favorable. Generally, absorbance at long wavelengths is enhanced due to developing shape anisotropy, the aggregation of small spherical NPs, or an increase in particle size [59,80,81].

## 4. Materials and Methods

### 4.1. Chemicals

All the chemicals were of analytic grade. 1,1-Diphenyl-2-picrylhydrazyl free radical (DPPH), ascorbic acid, and chlorogenic acid were purchased from Sigma-Aldrich (St. Louis, MO, USA). Methanol was obtained from Merck (Darmstadt, Germany). Water purified by an ULTRAPURE Millipore Direct-Q 3UV-R (Merck, Darmstadt, Germany) of the resistivity 18.2 MΩ cm was used to prepare all the aqueous solutions.

### 4.2. Collection of Plant Material and Sample Preparation

The fresh plants Aegopodium podagraria, Urtica dioica, Salvia officinalis, Levisticum officinale, Tilia cordata, Capsicum baccatum, and Viscum album were harvested in the southeastern region of Poland in May 2020. The roasted sea algae (Porphyra Yezoensis) used to make nori, Camellia sinensis, and Ilex paraguariensis were purchased from a local market. The fresh plants were dried for about three months in a shady area. These samples were then ground into a powder and subjected to extraction. For extraction, 5 g of powder was added to 100 mL of methanol. The samples were left for 60 min in an ultrasonic bath (ultrasound power 1200 W, frequency 35 kHz) Bandelin Sonorex RK 103 H (Bandelin Electronics, Berlin, Germany) at a temperature of 35 °C. The obtained extracts were centrifuged, and the supernatant was filtered through a syringe filter (0.7 µM or 0.45 µM) and stored in a refrigerator at 4 °C.

### 4.3. Reducing Antioxidant Power (CUPRAC)

The CUPRAC method is based on testing the ability of antioxidants to reduce copper(II) ions to copper(I). Commercially available kits enable the performance of microscale spectrophotometric measurements with 96-well plates. The assay measures total antioxidant capacity (TAC). The detection is based on a colored complex formed between the resulting Cu(I) and a dye reagent. The color intensity is proportional to TAC of the sample. The absorbance measurements for standards (ascorbic acid, chlorogenic acid, and Trolox) and extract samples in dilutions 1:1, 1:10, and 1:100 were performed in duplicate using Antioxidant Assay Kit-MAK334 purchased from Sigma-Aldrich Inc. (St. Louis, MO, USA). The 96-well microplate was mixed and incubated for 10 min at room temperature. The absorbance of extracts, reference chemicals, and blanks was measured at 570 nm by the use of a spectrophotometer Hybrid Multi-Mode Reader Synergy™ H1 (BioTek, Baden-Württemberg, Germany). Calibration curves were constructed for the reference substances. Linearity in the range from 300 to 1000 μM was achieved. The TAC values of the examined extracts were calculated according to the following equation:

(4) $$\mathrm{TAC}\;\left(\mu M\right)=\frac{{\left(A_{570}\right)}_{\mathrm{sample}}-{\left(A_{570}\right)}_{\mathrm{control}\;}}{\mathrm{Slope}\;\left({\mu M}^{-1}\right)}\;\times \;n$$

where (A_570_)_sample_ represents the absorbance of the sample, (A_570_)_blank_ is the absorbance of the medium blank, *n* stands for sample dilution factor, and Slope is related to linear calibration curve prepared for reference chemical.

### 4.4. DPPH Free Radical Scavenging Activity Assay

The stock solution of DPPH-R (1 mM) was prepared in methanol and stored in darkness at 4 °C in glass volumetric flasks. The working standard solutions were prepared daily by diluting the stock solution. The measurements were performed using spectrophotometer-Genesys 20 (The ThermoSpectronic, Waltham, MA, USA), which is able to measure absorbance in the range of 320–1100 nm. Spectrophotometric measurements were started with the selection of DPPH concentration so as to ensure absorbance on a level of 0.9 at 517 nm. The conducted experiments showed that this value corresponds to a concentration of 0.1 mM DPPH (Abs_control_). Thus, the total concentration of DPPH was kept at a constant level of 0.1 mM. In the first step, calibration curves were performed for solutions of ascorbic acid, chlorogenic acid, and Trolox as reference antioxidants. Measurements were performed for six different concentration levels after 15 min storage in a dark place. The calibration curves were subjected to simple linear regression analysis. The DPPH radical scavenging activity of extracts (Abs_sample_) was determined by measuring the absorbance of mixtures prepared by adding the different sample volumes to 1 mL of 1 mM methanolic solution of DPPH and made up to 10 mL with methanol. The measurements were performed after 1 h incubation in the dark at room temperature (20 °C). The DPPH radical scavenging activity of the samples was expressed as the percentage of inhibition (%I) calculated using the following equation:

(5) $$\%I=\frac{{\mathrm{Abs}}_{\mathrm{sample}}}{{\mathrm{Abs}}_{\mathrm{control}}}\times 100$$

### 4.5. The Silver NanoParticle Antioxidant Capacity (SNPAC)

The silver nanoparticle antioxidant capacity method was applied to evaluate the total antioxidant capacity (TAC) of the examined extracts. The SNPAC measurements were performed using the procedure described by Özyürek et al. [45]. The initial SNP solution (SNPs) was prepared by dropwise adding 5 mL of a 1% tripotassium citrate solution to 50 mL of 1 mM silver nitrate heated to 90 °C with constant stirring using a magnetic stirrer until a pale-yellow color. The resulting SNP solution was stored in the dark for 30 min. The test samples were prepared according to the following scheme: 2 mL of SNPs + x mL of 0.1 mM standard (ascorbic acid, Trolox, chlorogenic acid) or the tested extract + (0.8 − x) mL of water were mixed. After 30 min of storing in the dark, the mixtures turned to a dark brown color. The spectro-photometric measurements were made at 423 nm. The calibration curves were prepared in the range from 0 to 25 µM for ascorbic and chlorogenic acids or from 0 to 160 µM for the Trolox.

### 4.6. Synthesis of AgNPs

A measurement of 30 mL of 1 mM AgNO3 was heated for 15 min to approximately 90 °C. A total of 2 mL of the extract was then added dropwise with constant stirring using a magnetic stirrer. The finished solution was allowed to stand in the dark. The size of the silver nanoparticles and the zeta potential were measured with an analyzer (Litesizer, Anton PAAR, Graz, Austria) after 24 h, 72 h, and 96 h. UV-vis spectra were monitored as a function of the reaction time by a Genesys 20 (The ThermoSpectronic, Waltham, MA, USA) spectrophotometer operated at a resolution of 1 nm.

### 4.7. Fourier Transform Infrared (FTIR) Spectroscopy Measurements

Fourier transform infrared spectroscopy (FTIR) spectra were collected via a Nicolet 6700 FTIR spectrometer (Thermo Scientific, Waltham, MA, USA), with the Smart iTR attenuated total reflection (ATR, Thermo Scientific, Waltham, MA, USA) sampling accessory was used. Freeze dried samples were placed directly on ATR crystal and measured. The spectra were collected over the range of 4000–650 cm^−1^. For each material, 3 samples under the same conditions were examined. For each sample, 200 scans at a spectral resolution of 4 cm^−1^ were averaged. For a given material, the final average spectrum was then calculated. All spectral manipulation was carried out using Origin Pro 8.5 (OriginLab Corporation, Northampton, MA, USA).

### 4.8. SEM and EDS

Morphological forms and chemical composition of AgNPs occurrence were characterized by scanning electron microscopy (SEM) using Quanta 250 FEG Scanning Electron Microscope by FEI (Almelo, the Netherlands) equipped with energy dispersive spectrometry (EDS). Prior to SEM analysis, samples in liquid form were dried. Residues were dissolved in ethanol and the solvent was evaporated in a vacuum dryer at 70 °C. Then, the samples were glued to carbon tape on an aluminum holder and sputtered with graphite.

## 5. Conclusions

The current research proves that methanol extracts obtained from ten natural products, representatives of common herbs (*Salvia officinalis*, *Tilia cordata*, *Levisticum officinale*, *Aegopodium podagraria*, *Urtica dioica*, *Viscum album*), vegetables (*Capsicum baccatum*), marine algae (*Porphyra Yezoensis*), and teas (*Camellia sinensis*, *Ilex paraguariensis*), can be useful for the synthesis of AgNPs. Most of them have never been used for this purpose. Their free radical scavenging activity and reducing power were confirmed by DPPH, CUPRAC, and SNPAC assays. The DLS measurements showed different hydrodynamic diameters (99.56 to 119.68 nm) and the zeta potentials (−49.8 mV to −56.1 mV) of the obtained nanoparticles. It should be emphasized that the longer storage of nanoparticles in the reaction mixture promotes the adsorption of biomolecules, increasing the negative surface charge.

Considering the fact that phytochemical synthesis leads to the formation of nanoparticles with different morphological features, undergoing modification by active ingredients such as carboxylic acids, alcohol, phenol, esters, ethers, aldehydes, alkanes, and proteins, differences in pharmacological activity can be expected. Thus, research on the biological activity of synthesized NPs will be continued on bacterial strains, cell lines, and animal models.

## Figures and Tables

**Table 1:** Linear regression parameters for the calibration curves represented blank corrected absorbance vs. concentration [µM] of reference chemicals measured using Antioxidant Assay Kit (CUPRAC) at 570 nm.

Reference Chemical	Linearity Range (µM)	Slope ± SD	Intercept ± SD	Standard Error of Estimate (s_e_)	LOD (µM)	Fisher F Statistic (F)	Coefficient of Determination (R^2^)
Trolox	300–1000	0.00057 ± 0.00002	0.00331 ± 0.01470	0.018	86.84	624.29	0.9968
Chlorogenic acid	300–1000	0.00126 ± 0.00007	0.10900 ± 0.04400	0.055	115.23	332.97	0.9940
Ascorbic acid	300–1000	0.00065 ± 0.00002	−0.06010 ± 0.0127	0.016	65.48	646.32	0.9969

The REGLINP function was used to calculate the statistics for a straight line. The LOD was calculated from the following equations: LOD = (3.3 × σ/S), where the standard deviation of the response (σ) can be determined based on the standard deviation of y-intercepts of regression lines, whereas S represents the slope of the calibration curve.

**Table 2:** The values of the equivalents of the reference substances, i.e., Trolox, ascorbic acid, and chlorogenic acid for the tested extracts, calculated on the basis of calibration graphs made by the CUPRAC method with the Antioxidant Assay Kit.

Investigated Extracts	Absorbance [Mean Value] N = 2, dill. 1:100	TAC (mM)		
		Trolox	Ascorbic Acid	Chlorogenic Acid
*Aegopodium podagraria*	0.108	18.881	16.744	8.571
*Ilex paraguariensis*	0.271	47.377	42.015	21.507
*Porphyra Yezoensis* ^1^	0.114 ^1^	0.199 ^1^	0.176 ^1^	0.090 ^1^
*Urtica dioica*	0.005	0.874	0.775	0.396
*Camellia sinensis*	0.645	112.762	100.000	51.190
*Viscum album*	0.009	1.573	1.395	0.714
*Capsicum baccatum*	0.007	1.136	1.007	0.515
*Tilia cordata*	0.089	15.559	13.798	7.063
*Salvia officinalis*	0.152	26.573	23.565	12.063
*Levisticum officinale*	0.036	6.293	5.581	2.857

^1^ Absorbance result for undiluted extract.

**Table 3:** Linear regression parameters for the calibration curves representing the percentage of 0.1 mM DPPH absorbance inhibition vs. concentration (mM) of reference chemicals measured at 517 nm.

Reference Chemicals	Linearity Range (mM)	Slope ± SD	Intercept ± SD	Standard Error of Estimate (s_e_)	LOD (µM)	Fisher F Statistic (F)	Coefficient of Determination (R^2^)
Trolox	0.005–0.03	2757.764 ± 37.169	−1.1065 ± 0.7077	0.963	0.8498	5504.78	0.9993
Chlorogenic acid	0.01–0.025	2940.649 ± 95.967	−1.2686 ± 1.4789	1.730	1.6500	938.94	0.9978
Ascorbic acid	0.01–0.03	2756.221 ± 115.521	−3.3061 ± 2.2370	2.790	1.8044	569.26	0.9930

**Table 4:** The values of the equivalents of the reference substances, i.e., Trolox, ascorbic acid, and chlorogenic acid, for the tested extracts, calculated on the basis of calibration graphs made by the DPPH method.

Investigated Extracts	% I	Absorbance	TAC (mM)		
			Trolox	Ascorbic Acid	Chlorogenic Acid
*Aegopodium podagraria* ^2^	48.26	0.445	0.0614	0.1997	0.0578
*Ilex paraguariensis* ^1^	77.06	0.197	0.1252	0.4089	0.1177
*Porphyra Yezoensis* ^3,4^	25.84	0.709	0.0017	0.0054	0.0016
*Urtica dioica* ^2^	30.93	0.594	0.0446	0.1439	0,0420
*Camellia sinensis* ^1,5^	64.02	0.344	1.8893	6.1434	1.7763
*Viscum album* ^2^	20.23	0.631	0.0388	0.1249	0.0366
*Capsicum baccatum* ^2^	26.36	0.704	0.0276	0.0897	0.0259
*Tilia cordata* ^1^	62.09	0.326	0.1833	0.5958	0.1724
*Salvia officinalis* ^2^	78.26	0.187	0.1151	0.3755	0.1082
*Levisticum officinale* ^2^	30.23	0.600	0.0514	0.1666	0.0484

Sample volume/dilution: ^1^ 50 µL, ^2^ 100 µL, ^3^ 150 µL, ^4^ tightened 5×, ^5^ diluted 1:10.

**Table 5:** Linear regression parameters for the calibration curves of SNAPC method representing the absorbance measured at 423 nm vs. conc (µM) of reference chemicals.

Reference Chemical	Linearity Range (µM)	Slope ± SD	Intercept ± SD	Standard Error of Estimate (s_e_)	LOD (µM)	Fisher F Statistic (F)	Coefficient of Determination (R^2^)
Trolox	3.5–160.0	0.006189 ± 0.000177	0.067507 ± 0.01139	0.03013	5.8548	1224.18	0.9919
Chlorogenic acid	3.5–25.0	0.02975 ± 0.001567	0.0181 ± 0.02156	0.03307	2.5034	360.49	0.9863
Ascorbic acid	3.5–25.0	0.0169 ± 0.000571	0.0715 ± 0.007861	0.01206	1.5141	878.25	0.9943

**Table 6:** The values of the equivalents of the reference substances, i.e., Trolox, ascorbic acid, and chlorogenic acid, for the tested extracts, calculated on the basis of calibration curves made by the *SNAPC* method.

Investigated Extracts	Absorbance (Mean Value)	TAC (mM)		
		Trolox	Ascorbic Acid	Chlorogenic Acid
*Aegopodium podagraria* ^1^	1.172	9.9938	3.6460	2.1721
*Ilex paraguariensis* ^2^	1.244	53.2262	19.4260	11.5379
*Porphyra Yezoensis* ^1^	0.249	1.6422	0.5882	0.4346
*Urtica dioica* ^1^	0.899	7.5236	2.7420	1.6582
*Camellia sinensis* ^2^	1.696	73.6754	26.9148	15.7920
*Viscum album* ^1^	0.318	2.2665	0.8168	0.5645
*Capsicum baccatum* ^1^	0.495	3.8681	1.4033	0.8977
*Tilia mordata* ^2^	0.584	23.3668	8.4911	5.3261
*Salvia officinalis* ^2^	0.712	29.1577	10.6118	6.5308
*Levisticum officinale* ^1^	1.397	12.0296	4.3922	2.5956

sample volume: ^1^ 50 µL, ^2^ 10 µL.
